# Sex differences in the prediction of metabolic abnormalities via body mass index in an Eastern Chinese population

**DOI:** 10.3389/fnut.2025.1491818

**Published:** 2025-02-26

**Authors:** Yingjie Gao, Kaimeng Jin, Jin Chen, Ben Chen, Yan Guo, Jin Lu

**Affiliations:** Department of Endocrinology and Metabolism, Changhai Hospital, Naval Medical University, Shanghai, China

**Keywords:** body mass index, metabolic abnormalities, sex differences, Chinese population, health check-up

## Abstract

**Objective:**

Body mass index (BMI) is important for predicting the occurrence of metabolic abnormality, but sex differences exist. We aimed to investigate potential sex differences in the predictive value of BMI for metabolic abnormality and to calculate the optimal BMI cut-offs for predicting metabolic abnormality for each sex.

**Methods:**

Participants (*n* = 4,623) who attended a health check-up centre continuously in Eastern China between January 2022 and December 2023 were evaluated for metabolic abnormalities. We calculated the proportions of different metabolic abnormalities in different sexes. Receiver operating characteristic (ROC) curves were calculated to determine the optimal BMI cut-off values for predicting metabolic abnormality in males and females. The recognition rate of each metabolic abnormality using different BMI cut-off values for men and women were evaluated.

**Results:**

Among 4,623 participants (2,234 men and 2,389 women), the age-adjusted prevalence of all metabolic abnormalities was significantly greater among males than among females (*p* < 0.001). The optimal cut-off values for predicting metabolic abnormalities were 23.5 kg/m^2^ (males) and 21.8 kg/m^2^ (females). When BMI ≥24 kg/m^2^ was used as the cut-off value the recognition rates of each abnormal metabolic factor in the male group were all above 50%, while they were mostly below 50% in the female group, except for the recognition of hyperglycaemia and hypertriglyceridemia. However, in females, when BMI ≥22 kg/m^2^ was used as the cut-off value, the recognition rates for each abnormal metabolic factor were all above 50%, greater than that when BMI ≥24 kg/m^2^ was used (*p* < 0.001).

**Conclusion:**

There were sex differences in the BMI thresholds for predicting metabolic abnormalities in the health check-up population.

## Introduction

Obesity has been recognized by the World Health Organization (WHO) as a disease that can cause a series of health problems, such as increased risk of hypertension, diabetes, dyslipidaemia, coronary heart disease, myocardial infarction, stroke, tumours and other chronic diseases (1). Obesity can also lead to social and psychological problems, increasing the cost of health care for residents. Studies have shown that, in most individuals, body mass index (BMI) is strongly related to the amount of body fat and, compared to other measures, can better reflect the degree of obesity in the body. Throughout the world, the following BMI cut-off values established by the WHO are usually used: a BMI of 18.5–23.9 is considered to indicate normal weight, 25.0–29.9 is considered to indicate overweight status, and ≥30 is considered to indicate obesity status (2). China currently recommends using BMI cut-offs of ≥24.0 kg/m^2^ and ≥28.0 kg/m^2^ to diagnose overweight status (24.0 kg/m^2^ ≤ BMI < 28.0 kg/m^2^) and obesity status (BMI ≥28.0 kg/m^2^), respectively, in adults (3).

However, there are key aspects of metabolic homeostasis that are regulated differently in males and females (4–6), and it has been reported that there are sex differences in the prevalence of different combinations of metabolic syndrome (MetS) factors (7, 8). In addition, the association between ageing and MetS risk was shown to be stronger in females, while the association between unhealthy lifestyles and MetS risk was shown to be stronger in males (9). Interestingly, high-intensity housework was found to be positively associated with metabolic markers in women; however, this relationship was not observed in men (10, 11). An increased BMI is considered the main risk factor for MetS (5, 6, 12, 13), but few studies have focused on sex differences in the BMI cut-off value used to predict the risk of various metabolic disorder in individuals living in China. Furthermore, the prevalence of hyperuricemia is increasing and is associated with both cardiovascular disease and insulin resistance (14). The aim of this cross-sectional study was to investigate whether the BMI value used to predict metabolic abnormalities differs between males and females and to calculate the optimal predictive BMI value for each sex.

## Materials and methods

### Participants

In the present study, we recruited 4,623 people aged 18–70 years who voluntarily visited the Medical Examination Center of Eastern China for a health checkup between January 2022 and December 2023. All participants were of Han ethnicity. The exclusion criteria were as follows: (i) had evidence of liver or renal insufficiency or malignancy; (ii) had a medication history of corticosteroids or hormone therapy in the previous 6 months; (iii) participated in a weight-loss programme or had lost ≥5% of their body weight in the previous 12 months; (iv) had skeletal deformities, amputations or dependence on wheelchairs or other ambulatory assistive devices; and (v) were pregnant.

The study was approved by the Ethics Committee of the Shanghai Changhai Hospital (CHEC2024-263).

### Physical examination

Trained physicians administered a standard questionnaire to collect information on age, weight status, medical history and medication use. Routine physical examinations were then performed for all participants. Two blood pressure (BP) recordings (rounded to the nearest 2 mmHg) were obtained from the right arm of the participants after 30 min of seated rest. The final measurement was the average of these two recordings. Participants were asked to wear light clothing and take off their shoes for the measurement of their anthropometric characteristics, which was performed by well-trained examiners. Height was measured (rounded to the nearest 0.1 cm) using a portable stadiometer. With the participants in an upright position, body weight was measured (rounded to the nearest 0.1 kg) using a calibrated scale. BMI was calculated by dividing participants’ body weight by the square of their height (kg/m^2^).

### Biochemical measurements

After an overnight fast of 10–12 h, all participants provided blood samples, which were collected from a peripheral vein. After collection, the samples were immediately centrifuged at 4°C. The plasma lipid profile [including total cholesterol (TC), triglycerides (TG), low-density lipoprotein-cholesterol (LDL-C), and high-density lipoprotein-cholesterol (HDL-C) concentrations] and uric acid (UA) and fasting plasma glucose (FPG) concentrations were assayed using an automated analyser (Olympus AU5800, Japan).

### Definition of metabolic disorders

Metabolic data for 4,623 people (2,234 males and 2,389 females) were obtained from the Chinese Physical Examination Center. In the present study, the abnormal metabolic factor criteria were as follows: (1) systolic blood pressure (SBP) ≥130 mmHg; (2) diastolic blood pressure (DBP) ≥85 mmHg; (3) TG ≥1.7 mmol/L; (4) HDL-C <1.04 mmol/L; (5) TC ≥5.2 mmol/L; (6) LDL-C ≥3.4 mmol/L; (7) FPG ≥6.1 mmol/L; and (8) UA ≥420 μmol/L (males) or ≥360 μmol/L (females). Individuals who met at least 3 of the 8 above criteria were considered to have metabolic abnormalities, and the participants were divided into two groups: a metabolically normal group and a metabolically abnormal group.

### Statistical analyses

All analyses were performed with IBM SPSS Statistics software version 26.0 (Chicago, IL, United States). All continuous data are reported as the mean ± standard deviation (SD) and were tested for normality using the Kolmogorov–Smirnov test. If the data were not normally distributed, the Mann–Whitney *U* test was used for between-group comparisons. Partial correlation coefficients were calculated to assess the correlations between BMI and metabolic variables, including SBP, DBP, and concentrations of FPG, TC, TG, HDL-C, LDL-C, and UA, while controlling for age. The categorical variables are expressed as *n* (%) and were compared using the chi-square test. Receiver operating characteristic (ROC) curves were calculated to determine the best BMI cut-off values for predicting metabolic abnormalities. ROC curves were created using GraphPad Prism version 10.0.2 (GraphPad Software, San Diego, CA, United States). The recognition rates of metabolic abnormalities in the male and female groups were calculated separately under the different theoretical BMI cut-off values, and the results are expressed as *n* (%) (e.g., the recognition rate of male TC abnormalities = the number of males with TC ≥5.2 mmol/L and BMI ≥24/the number of males with TC ≥5.2 mmol/L). *p* < 0.05 was considered to indicate statistical significance.

## Results

### Comparison of metabolic indexes and the prevalence of metabolic abnormalities between men and women

Overall, this study included 4,623 participants (2,234 men and 2,389 women). The mean age was 35.12 ± 10.72 (18–69) years. We found significant between-sex differences in all variables (Table 1). Compared with men, women were younger; had a lower BMI; had lower FPG, TC, TG, LDL-C, and blood pressure; and had higher levels of HDL-C (all *p* < 0.01).

**Table 1:** Clinical and biochemical variables of all participants according to sex.

Variables	All participants *N* = 4,623	Males *N* = 2,234	Females *N* = 2,389	*p*
AGE	35.12 ± 10.72	36.05 ± 10.91	34.24 ± 10.47	0.000
BMI (kg/m^2^)	22.67 ± 3.47	24.08 ± 3.43	21.36 ± 2.95	0.000
FPG (mmol/L)	5.07 ± 0.85	5.20 ± 1.01	4.95 ± 0.63	0.000
TC (mmol/L)	4.48 ± 0.87	4.55 ± 0.86	4.42 ± 0.87	0.000
TG (mmol/L)	1.33 ± 1.03	1.64 ± 1.19	1.03 ± 0.73	0.000
HDL-C (mmol/L)	1.37 ± 0.36	1.21 ± 0.30	1.52 ± 0.34	0.000
LDL-C (mmol/L)	2.72 ± 0.75	2.61 ± 0.73	2.43 ± 0.76	0.000
SBP (mmol/L)	111.93 ± 16.13	117.40 ± 15.19	106.81 ± 15.29	0.000
DBP (mmol/L)	67.84 ± 11.05	71.53 ± 10.93	64.39 ± 9.99	0.000
UA (μmol/L)	334.47 ± 91.06	396.61 ± 79.23	276.37 ± 56.49	0.000

BMI, body mass index; FPG, fasting plasma glucose; TC, total cholesterol; TG, triglyceride; HDL-C, high-density lipoprotein-cholesterol; LDL-C, low-density lipoprotein-cholesterol; SBP, systolic blood pressure; DBP, diastolic blood pressure; UA, uric acid. Difference between males and females was tested.

We further calculated the proportions of different metabolic abnormalities in different sexes. When stratified by sex, 1,111 (49.7%) males and 407 (17.0%) females had overweight status (Table 2), 495 (22.2%) males and 211 (8.8%) females had hypertension (SBP ≥130 mmHg or DBP ≥85 mmHg), 148 (6.6%) males and 44 (1.8%) females had hyperglycaemia (FPG ≥6.1 mmol/L), 1,176 (52.6%) males and 581 (24.3%) females had dyslipidaemia (TC ≥5.2 mmol/L, TG ≥1.7 mmol/L, HDL-C <1.04 mmol/L, and LDL-C ≥3.4 mmol/L), and 787 (35.2) males and 171 (7.2) females had hyperuricaemia [UA ≥420 μmol/L (males) or UA ≥360 μmol/L (females)]. The age-adjusted prevalence of each abnormal metabolic factor in the male group was greater than that in the female group (*p* < 0.001) (Table 2).

**Table 2:** Prevalence of each metabolic abnormality between men and women.

Metabolic abnormalities	All participants *N* = 4,623 (%)	Males *N* = 2,234 (%)	Females *N* = 2,389 (%)	*p*
Overweight (BMI ≥24)	1,518 (32.8)	1,111 (49.7)	407 (17.0)	0.000
SBP ≥130 or DBP ≥85 mmHg	706 (15.3)	495 (22.2)	211 (8.8)	0.000
Hyperglycaemia (FPG ≥6.1 mmol/L)	192 (4.2)	148 (6.6)	44 (1.8)	0.000
TC ≥5.2 mmol/L	844 (18.3)	470 (21.0)	374 (15.7)	0.000
TG ≥1.7 mmol/L	959 (20.7)	743 (33.3)	216 (9.0)	0.000
HDL-C <1.04 mmol/L	836 (18.1)	684 (30.6)	152 (6.4)	0.000
LDL-C ≥3.4 mmol/L	511 (11.1)	308 (13.8)	203 (8.5)	0.000
UA ≥420 μmol/L (males)	958 (20.7)	787 (35.2)	171 (7.2)	0.000
UA ≥360 μmol/L (females)				

BMI, body mass index; SBP, systolic blood pressure; DBP, diastolic blood pressure; FPG, fasting plasma glucose; TC, total cholesterol; TG, triglyceride; HDL-C, high-density lipoprotein cholesterol; LDL-C, low-density lipoprotein cholesterol; UA, uric acid. Difference between males and females was tested.

### BMI cut-off values for predicting metabolic abnormalities in men and women

Further analysis of the association between BMI and metabolic abnormalities by sex revealed that after controlling for age, BMI was positively correlated with SBP, DBP, and the concentrations of FPG, TC, TG, LDL-C, and UA and negatively correlated with the serum HDL-C concentration in both males and females (Table 3).

**Table 3:** Partial correlations of BMI with metabolic variables after controlling for age.

	BMI					
	All participants *N* = 4,623		Males *N* = 2,234		Females *N* = 2,389	
	*r*	*p*	*r*	*p*	*r*	*p*
SBP (mmol/L)	0.332	0.000	0.260	0.000	0.200	0.000
DBP (mmol/L)	0.314	0.000	0.274	0.000	0.168	0.000
FPG (mmol/L)	0.200	0.000	0.161	0.000	0.181	0.000
TC (mmol/L)	0.186	0.000	0.230	0.000	0.103	0.000
TG (mmol/L)	0.376	0.000	0.322	0.000	0.266	0.000
HDL-C (mmol/L)	−0.456	0.000	−0.370	0.000	−0.330	0.000
LDL-C (mmol/L)	0.223	0.000	0.214	0.000	0.165	0.000
UA (μmol/L)	0.497	0.000	0.371	0.000	0.305	0.000

Pearson’s correlation coefficient; SBP, systolic blood pressure; DBP, diastolic blood pressure; TG, triglyceride; TC, total cholesterol; HDL-C, high-density lipoprotein cholesterol; LDL-C, low-density lipoprotein cholesterol; FPG, fasting plasma glucose; UA, uric acid.

**Figure 1:**
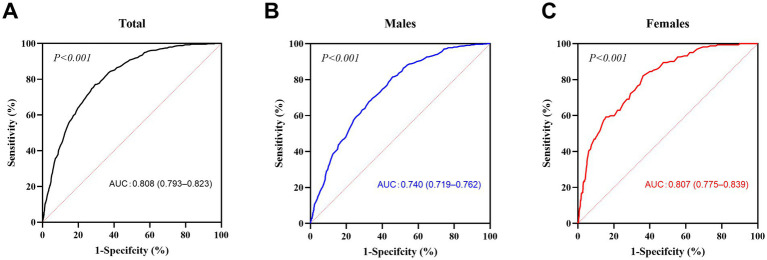
ROC curves for BMI. The receiver operating characteristic (ROC) curves of body mass index (BMI) are presented for all participants (**A**, *n* = 4,623), males (**B**, *n* = 2,234) and females (**C**, *n* = 2,389). The dotted line is the baseline (minimum standard), and the solid line is the ROC curve. *p* < 0.05 was considered to indicate statistical significance.

Of the 4,623 participants, 789 (17.1%) were in the metabolically abnormal group, and 3,834 (82.9%) were in the metabolically normal group. The areas under the ROC curves (AUCs) were calculated to determine the predictive value of BMI for metabolic abnormalities (Figure 1). The AUCs for BMI were 0.808 [95% confidence interval (CI), 0.793–0.823] for all participants combined, 0.740 (95% CI, 0.719–0.762) for males and 0.807 (95% CI, 0.775–0.839) for females. We calculated the Youden index and identified BMI ≥23.5 kg/m^2^ (sensitivity 81.5%, specificity 54.3%) and BMI ≥21.8 kg/m^2^ (sensitivity 82.1%, specificity 64.3%) as the best cut-off values for identifying metabolic abnormalities in males and females, respectively.

At BMI thresholds of 20, 22, and 24 kg/m^2^, sensitivity and specificity varied significantly between sexes (Supplementary Table S1). For females, a lower threshold of 22 kg/m^2^ achieved higher sensitivity (82.1%) compared to 24 kg/m^2^ (57.4%), aligning with the optimal cut-off identified by the Youden index. In contrast, for males, the traditional threshold of 24 kg/m^2^ maintained reasonable sensitivity (81.5%) while balancing specificity (54.3%). These findings highlight the need for sex-specific BMI criteria in metabolic risk screening.

### Recognition rate of metabolic abnormalities using different BMI cut-off values for men and women

**Figure 2:**
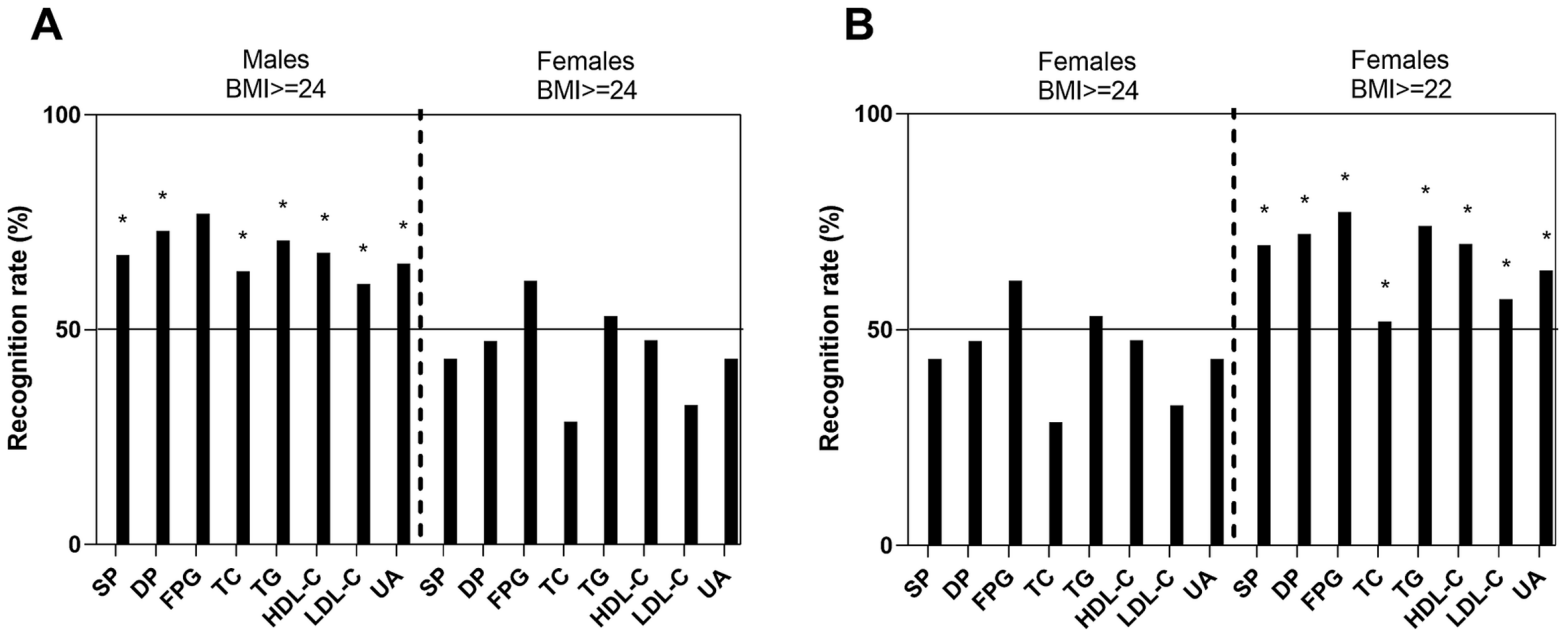
Recognition rates of metabolic abnormalities with a BMI ≥24 as the cut-off value for both men and women **(A)** and with different BMI cut-off values (≥24 or ≥22, respectively) for men and women **(B)**. The black bars represent the recognition rate of abnormal values for each metabolic index under the different theoretical BMI cut-off values according to sex. ^*^p < 0.01 between groups. SP, systolic blood pressure; DP, diastolic blood pressure; FPG, fasting plasma glucose; TC, total cholesterol; TG, triglyceride; HDL-C, high-density lipoprotein cholesterol; LDL-C, low-density lipoprotein cholesterol; UA, uric acid.

We then used the above two BMI cut-off points to calculate the recognition rates of each metabolic abnormalities among males and females. When BMI ≥24 kg/m^2^ was used as the screening standard, the recognition rates of each abnormal metabolic factor in the male group were all above 50%, while they were mostly below 50% in the female group, except for the recognition of hyperglycaemia (FPG ≥6.1 mmol/L) and TG ≥1.7 mmol/L. Most recognition rates in the male group were greater than those in the female group (*p* < 0.001), except for the prediction of hyperglycaemia (FPG ≥6.1 mmol/L) (*p* > 0.05) (Figure 2A). However, in females, when BMI ≥22 kg/m^2^ was used as the screening standard, the recognition rates for each abnormal metabolic factor were all above 50%, greater than that when BMI ≥24 kg/m^2^ was used (*p* < 0.001) (Figure 2B). Notably, there was no difference in the recognition rate of metabolic abnormalities between women with a BMI ≥22 kg/m^2^ and men with a BMI ≥24 kg/m^2^ (*p* > 0.05). Different BMI cut-off points yield substantial differences in the prediction of metabolic abnormalities in men and women. Using a cut-off of BMI ≥22 has the same prediction effect on women as a cut-off of BMI ≥24 did for men.

## Discussion

In this study, we collected data from participants who attended a health check-up centre continuously in Eastern China and analysed the occurrence of various metabolic abnormalities. Consistent with previous studies, correlations between BMI and different metabolic abnormalities were observed. At the same time, we calculated the BMI prediction cut-off point by sex and observed similar predictive effectiveness for metabolic abnormalities in men with BMI ≥24 and women with BMI ≥22.

BMI, which is widely used in clinical work and epidemiological studies, is also an important indicator for the diagnosis of obesity. The present study contributes to the body of literature by further elucidating the sex differences in the relationship between BMI and different metabolic abnormality phenotypes. Our study revealed a significantly greater prevalence of overweight, hypertension, hyperglycaemia, dyslipidaemia, and hyperuricaemia in males than in females. These results are consistent with those of most studies (9, 15–17). However, other studies have shown that the prevalence of these metabolic abnormalities is greater in females than in males (6, 18, 19). The different results among the studies may be explained by differences in hormones, lipid metabolism, population diversity, cultural behaviors, lifestyle habits, and the use of different diagnostic criteria. Some research has shown that sex may be an independent predictor of differences in most metabolic components. Although hypertension, prediabetes, and hypertriglyceridemia are more common in males than in females, the prevalence of low HDL and high WC is greater in females than in males (6, 20). However, most previous related studies (21, 22) have shown that metabolic components, including diabetes and hypertension, confer a greater risk for CVD in women than in men. The sex difference in the prevalence of metabolic components, at least in part, can be attributed to differences in dysglycaemia, body fat, adipocyte biology, and hormonal control of body weight (10, 11, 23).

Although the burden of MetS and its components has been increasing mainly among male individuals, clinical outcomes related to MetS (i.e., cardiovascular diseases) are worse among female individuals (24). Whether the existing predictors of MetS (i.e., BMI) differ by sex is a matter of debate. A WHO expert consultation concluded that the cut-off point for observed risk varies from 22 kg/m^2^ to 25 kg/m^2^ in different Asian populations; for high risk, the BMI cut-off varies from 26 kg/m^2^ to 31 kg/m^2^, and the WHO proposed methods by which countries could make decisions about the definitions of increased risk for their population (25). In this study of 4,623 Chinese adults, we found that a BMI threshold of 23.5 kg/m^2^ was considered the best cut-off value for detecting metabolic abnormalities in the male group, while 21.8 kg/m^2^ was considered the best cut-off value in the female group. We then evaluated the two BMI cut-off points for predicting abnormal metabolism in the male and female groups and found that when the BMI was ≥24, the rate of metabolic abnormalities in the male subgroup was greater than that in the female subgroup, except for the recognition of hyperglycaemia. In the female group, the BMI threshold of 22 kg/m^2^ was more efficient than that of 24 kg/m^2^ as the screening standard for metabolic abnormalities. However, BMI should not be used as a diagnostic indicator yet and needs to be validated in another large population sample. It is also still necessary to comprehensively judge metabolic abnormalities based on indicators such as waist circumference, blood lipids, blood sugar, body fat percentage, eating habits, and family history.

In studies conducted in Turkey (26) and India (27), the differences in BMI distribution between men and women older than 20 years were analysed, and the difference was mainly due to differences in sociodemographic and behavioral factors. Fruzzetti et al. (28) reported that BMI, sex hormone binding globulin (SHBG), and the free androgen index were superior predictors of metabolic abnormalities in women with polycystic ovary syndrome (PCOS). The cut-offs that emerged from their curves ranged from 25.05 to 35.15 kg/m^2^, indicating that overweight status (BMI ≥25 kg/m^2^) is already a risk factor for initial metabolic damage. Therefore, it is reasonable that the BMI cut-off is much lower for healthy women, as in our study, than for women with PCOS. Another cross-sectional study in China (29) revealed that in women, obesity was negatively and significantly associated with health-related quality of life (HRQOL), whereas in men, this association was positive but not statistically significant. A possible explanation for sex differences in obesity is that women might be more susceptible to distress related to body weight or body image than men are (30, 31). Excessive dieting may improve other metabolic abnormalities, such as blood pressure, blood glucose and lipid profiles, in obese women. This could also be explained by cultural perceptions about personal body weight and more discrimination against women with excess body weight in their work-related life and social roles (31, 32).

However, BMI has various pitfalls as a measure of obesity, especially when BMI is based on self-reported height and weight. BMI does not necessarily reflect changes with age. The proportion of body fat increases with age, while muscle mass decreases, but corresponding changes in height, weight, and BMI may not reflect changes in body fat and muscle mass. The prediction of body fat in elderly individuals is not as effective as that in young and middle-aged individuals (33). Some Asian populations (including Chinese populations) have greater body fat percentages and health risks than Caucasians for a given BMI (34). There are sex and age differences, even inverse correlations, in the relationships of BMI with body fat mass and percentage, especially in boys before and after puberty (35). The strength of our data analysis study is that the influence of younger and older age groups (i.e., minors and elderly individuals older than 70 years of age) on BMI was excluded. There was no restriction on the screening of the population, and as many participants as possible were included to obtain objective statistical results. The male and female groups were skewed and distributed approximately the same. We used analysis of covariance to eliminate the difference in age and compared the male and female groups, and the results were still statistically significant.

This study also has many limitations. The study applied a cross-sectional design, so causal relationships cannot be determined. The study participants mainly came from the eastern region of China, which may not fully represent the metabolic characteristics of other regions or populations. Therefore, the cut-off values proposed herein should be further validated. Additionally, using BMI as an indicator to assess obesity also has limitations, and this study did not consider other potential metabolic abnormality prediction factors (such as waist circumference, body fat percentage). Finally, the recognition rate also cannot effectively verify the validity of the BMI cut-off value. Further research with a larger sample is necessary.

In conclusion, the purpose of this study was to demonstrate that the accuracy of BMI cut-offs for the prediction of metabolic abnormalities differed by sex. In females, compared to a BMI cut-off of ≥24, a BMI cut-off of ≥22 may be a more useful predictor of metabolic abnormalities. BMI can be used as an easy predictor of metabolic abnormalities without the need for a hospital visit. Our findings reinforced the sex differences in the BMI cut-off predictor value for MetS and its risk factors, which has implications for the future development of sex-specific MetS prevention and intervention programs.

## Data Availability

The data analyzed in this study is subject to the following licenses/restrictions: the datasets used and analysed during the current study are available from the corresponding author upon reasonable request. Requests to access these datasets should be directed to guoyansmmu@hotmail.com.
