# Supplementary material for: Sex differences in the prediction of metabolic abnormalities via body mass index in an Eastern Chinese population
Source: Front Nutr. 2025 Feb 26;12:1491818. doi: 10.3389/fnut.2025.1491818 (PMC11896866; doi:10.3389/fnut.2025.1491818)
Supplement: Supplementary file 1 [file Table_1.pdf]

Supplementary Materials

Table S1. The sensitivity and specificity values for BMI thresholds of 20, 22, and 24 kg/m² in males and females.

Sex	BMI (kg/m²)	Sensitivity%	95% CI	Specificity%	95% CI	Likelihood ratio
**Male**	20.0	98.72	97.50% to 99.35%	14.69	13.04% to 16.50%	1.157
**Male**	23.5*	81.50	78.27% to 84.34%	54.32	51.88% to 56.75%	1.784
**Male**	24.0	74.64	71.09% to 77.89%	59.99	57.57% to 62.36%	1.865
**Female**	20.0	95.06	90.56% to 97.48%	36.03	33.93% to 38.18%	1.486
**Female**	21.8*	82.10	75.47% to 87.24%	63.77	61.61% to 65.87%	2.266
**Female**	24.0	57.41	49.71% to 64.76%	85.67	84.05% to 87.15%	4.006

Abbreviations: * The optimal BMI cut-off values were determined using the Youden index.
